# Association between freedom of movement and health of nursing home residents with dementia: an exploratory longitudinal study

**DOI:** 10.1186/s12877-024-04677-z

**Published:** 2024-02-26

**Authors:** Suzan van Liempd, Sascha Bolt, Marjolein Verbiest, Katrien Luijkx

**Affiliations:** 1 Department of Tranzo, Scientific Centre for Care and Wellbeing, School of Social and Behavioral Sciences, Tilburg University, Tilburg, The Netherlands; 2 Stichting Mijzo, Waalwijk, The Netherlands

**Keywords:** Freedom of movement, Open doors, Nursing homes, Dementia, Positive health

## Abstract

**Background:**

Locked doors remain a common feature of dementia units in nursing homes (NHs) worldwide, despite the growing body of knowledge on the negative effects of restricted freedom on residents. To date, no previous studies have explored the health effects of opening locked NH units, which would allow residents to move freely within the building and enclosed garden. This study examines the association between increased freedom of movement and the health of NH residents with dementia.

**Methods:**

This longitudinal, pre-post study involved a natural experiment in which NH residents with dementia (*N* = 46) moved from a closed to a semi-open location. Data on dimensions of positive health were collected at baseline (T0; one month before the relocation), at one (T1), four (T2) and nine (T3) months after the relocation. Linear mixed models were used to examine changes in positive health over time.

**Results:**

Cognition, quality of life and agitation scores improved significantly at T1 and T2 compared to the baseline, while mobility scores decreased. At T3, improvements in agitation and quality of life remained significant compared to the baseline. Activities of daily living (ADL) and depression scores were stable over time.

**Conclusions:**

Increasing freedom of movement for NH residents with dementia is associated with improved health outcomes, both immediately and over time. These findings add to the growing evidence supporting the benefits of freedom of movement for the overall health of NH residents with dementia.

## Background

When living at home is no longer safe or when care needs exceed the available support at home, people with dementia often move into a nursing home (NH). In a NH, residents often have complex care needs and increased vulnerability, and they require assistance with activities of daily living (ADLs). NHs provide 24-hour functional support and care for residents [1]. About 290.000 people with dementia live in the Netherlands of whom 80.000 live in a care facility [2]. NH residents with dementia often live in special care units [3]. Many of these units have locked entrance doors to protect residents with dementia from potential harm when wandering out [4]. It is estimated that 50.000 people with dementia in the Netherlands live in a closed unit [5].

Keeping people behind locked doors limits their freedom of movement [6]. Freedom of movement can be defined as the right to decide to independently move from one place to another [7]. There are different levels of freedom of movement within a nursing home setting. In closed NHs, residents with dementia are free to move within a care unit but are not allowed to independently leave their unit without supervision. In semi-open NHs, residents have freedom of movement within the NH building and/or enclosed gardens but are not allowed to enter the outside world independently. In an open NH, residents are free to go wherever they like, and they are not hindered by closed entrance doors [7]. A recent review of the literature showed that the level of freedom of movement in NHs can be related to the health of residents with dementia [7]. There has been a shift from health defined as the absence of physical disease towards a more holistic view on health, which focuses on the whole person [8]. The concept of positive health incorporates this holistic view on health, encompassing six dimensions: (1) physical health, (2) quality of life, (3) existential health, (4) social and societal participation, (5) mental functions and perception and (6) daily living [9].

Restrictions on freedom by means of locked doors may infringe upon resident autonomy [10]. When hindered by locked entrance doors, residents could experience a sense of captivity related to their limited opportunities to go outside [11]. Other negative health outcomes that could arise in relation to being kept behind locked doors include frustration and agitation [12] and feelings of being controlled [13]. Recent studies on the impact of Covid-19 restrictions showed that prolonged isolation directly increases the risk of neuropsychiatric symptoms and severe behavioral disturbance among NH residents with dementia [14]. People with dementia value freedom and choice [15], including having free access to outdoor spaces and nature [13]. Increased freedom of movement of NH residents has been shown to relate to lower levels of agitation and higher quality of life compared with residents living in locked units [12, 16].

Many NHs still have dementia units with locked doors, despite the potential negative effects on residents’ health. The main arguments for upholding restrictions on freedom of movement involve safety and risk management, which often outweigh arguments related to individual needs and the autonomy of residents [10, 17]. To support NHs in creating a care environment that is appropriate for persons with dementia, it is therefore important to further study associations between freedom of movement and the health of NH residents with dementia.

Studies on freedom of movement and health often used a qualitative design and therefore do not provide information on associations between freedom of movement and health benefits over time [7]. Moreover, most studies focused on single aspects of health, such as mental well-being or quality of life. To date, only a few longitudinal studies have investigated whether and how increasing freedom of movement in an NH is associated with the health of residents with dementia [16, 18, 19]. In these studies, increasing freedom of movement was limited to adding a freely accessible garden. No previous studies have explored the effects of opening locked units within a NH to allow residents to move freely within the building and enclosed garden. Additionally, studies on the association of freedom of movement with the dimensions of positive health for NH residents with dementia are lacking altogether. Freedom of movement of people with dementia in long-term NH care may relate to these distinct health dimensions [7]. Therefore, in this study, we operationalize health using the concept of positive health [20]. The aim of this exploratory study is to investigate whether and to what extent increased freedom of movement is associated with the positive health of NH residents with dementia over time.

## Methods

This study involved a natural experiment in which a group of NH residents with dementia was observed before and after a relocation between NHs in September 2020. The residents moved from a closed NH with dementia units with locked doors to a semi-open NH with freedom of movement within the entire building and free garden access. Their own belongings were transferred to the new location, and the entire team of care professionals also remained the same. A longitudinal study design with four repeated measures was used to examine the associations between increased freedom of movement and the dimensions of positive health over time. Data were collected at baseline (T0; one month before the relocation), at one (T1), four (T2) and nine (T3) months after the relocation. Data collection took place between August 2020 and June 2021.

### Settings

The closed NH and the semi-open NH are two separate facilities owned by the same non-profit, publicly funded long-term care organization in the Netherlands. Both facilities fit the international definition of an NH: “a facility with a domestic environment that provides 24-hour functional support and care for persons who require assistance with ADLs and who often have complex health needs and increased vulnerability” [1].

The closed NH housed 50 residents with dementia and 25 residents who needed both gerontopsychiatric and physical care. Residents with dementia lived in four separate locked units. Each unit had its own common living room, and all residents had their own private apartment. The units were located on the first, second and third floors, and unit doors were locked with security codes. Residents were not allowed to leave the unit independently. The building had an enclosed garden with a terrace, vegetable garden and chickens. Residents with dementia were allowed to access the garden only under supervision.

The semi-open NH is a newly built facility (2020) with capacity of 75 residents: 55 residents with dementia and 20 residents who need both gerontopsychiatric and physical care. The care concept differs from the closed NH because there are no separate unit doors. All residents with dementia are free to move around within the building and surrounding enclosed gardens with different terraces, benches, chickens and a bird cage. The first floor includes seven community rooms, each with a specific theme, positioned on a large square with a kiosk. The residents’ apartments are situated on the second and third floors. Thereby, residents are encouraged to move from their apartment to the first floor every day, and they can decide for themselves where they want to go. Technological applications, such as wristbands with wander detection, are used to prevent residents from going outside the front door of the building or outside the garden without supervision.

We used the OAZIS-Dementia to describe the physical differences between the two settings (closed and semi-open NH) on eight categories, including the following: 1) privacy and autonomy (11 items): the extent to which residents’ privacy is safeguarded and respected, 2) comfort and control (7 items): the degree of comfort of the environment and the extend to which residents themselves can adapt and influence their environment according to their own preferences, 3) windows and views (10 items): the extent of daylighting and views for residents, 4) facilities (8 items): the availability of facilities and activities of interest to residents, 5) orientation and routing (14 items): the extent to which a clear and understandable layout of the building is provided, 6) interior (11 items): the extent to which the interior and furnishings match residents’ frames of reference, 7) nature (7 items): the extent to which residents can have direct or indirect contact with nature, and 8) staff (9 items): the extent to which a building is aligned with staff work and care processes [21]. OAZIS stands for ‘Onderzoek Aantrekkelijkheid Zorgomgevingen met behulp van de Impact Scan’ (translated: Research Attractiveness Healthcare Environments using the Impact Scan) and is a tool to gain insight into relevant environmental characteristics of a NH for determining the attractiveness of these environments. A pilot test in three NHs during the development of the instrument demonstrated a high inter-rater reliability of the OAZIS-Dementia [22]. For both settings, the facilities’ team manager, care manager and a care professional completed the OAZIS-Dementia together in which disagreements between them were discussed until consensus was reached. After completing the OAZIS-Dementia, the program automatically generated the mean scores for each category.

### Sample

In total, 75 residents lived in the closed NH, of which 55 residents had a diagnosis of dementia and were therefore eligible for inclusion. They were (almost) completely dependent on care, based on their care intensity package. This is a Dutch proxy for the intensity of NH care that the resident needs which is assessed by the Care Needs Assessment Centre [23]. Legal representatives of all residents with dementia received written information about the study from the researcher and were asked to provide written consent. At baseline, the researcher (SvL) gathered the demographic characteristics of residents for whom permission had been obtained from legal representatives through a care-record review. These characteristics included sex, age and type of dementia. The researcher (SvL) followed a short training by the organization’s application administrator to be able to consult the care records of residents in a correct and careful manner.

### Health outcomes

Resident health was operationalized using the six dimensions of the positive health model [9]. Different questionnaires were used to measure the residents’ health on these dimensions. First, quality of life and participation were measured using the Qualidem [24]. The Qualidem contains nine subscales, which are all applicable to people with dementia [25]. The reliability is good to excellent for the subscales positive affect, positive self-image, care relationship and negative affect, questionable–acceptable for restless tense behaviour, social relations, social isolation and feeling at home, and poor for having something to do [25]. Second, mental functioning and perception were measured using the Cohen-Mansfield Agitation Inventory (CMAI) [26], the Cornell Scale for Depression in Dementia (CSDD) [27] and the InterRAI Long-Term Care Facilities System (interRAI LTFC) section C, Cognition [28]. The CMAI is considered a valid and reliable scale to assess agitation [29]. The CSDD is considered a reliable instrument for use in NH residents with dementia [30] and has demonstrated good accuracy [31]. The interRAI LTCF is an independent geriatric assessment system for the key domains of health and function of older people, including cognition, and it has demonstrated adequate reliability across various long-term settings [32]. Third, daily functioning was assessed with the Barthel Index [33]. For assessing residents with dementia, the internal consistency was acceptable [34]. Fourth, bodily functions, in particular balance and mobility, were measured using the Performance-Oriented Mobility Assessment according to Tinetti (POMA) [35], with an acceptable predictive validity concerning fall risk [36]. The inter-rater reliability of the instrument was good [36]. No appropriate questionnaire was found to address the existential dimension. In this study we chose to align as much as possible with existing practices in the organization, to minimize the burden on care professionals in terms of their contribution to the study. The CMAI, CSDD and POMA instruments are routinely used in the participating organization. During each measurement, the same two care professionals per unit, who were closely involved in the care of the included residents, completed in pairs the Qualidem, CMAI, InterRAI LTFC section C and Barthel Index for each resident. Any discrepancies between the two care professionals were resolved through discussion, until consensus was reached. Upfront, all care professionals were carefully instructed by the researcher to establish uniformity in the data collection.

The questionnaires were combined into one online survey using Qualtrics [37] to minimize the burden on care professionals. The questionnaires were completed digitally using a laptop. The CSDD was, conform the questionnaire completion instructions, administered for each resident at every time point by a psychologist and care professional per team and registered in the care record of the resident. A physical therapist administered the POMA to each resident who could cognitively and physically perform the test and registered the results in the care record. The researcher (SvL) exported the results from the care record into Qualtrics. All demographic and questionnaire data from Qualtrics were exported to SPSS for analysis.

### Analysis

Statistical analyses were carried out using SPSS version 28 for Windows. To describe the demographic characteristics of the respondents, we calculated their mean age and standard deviation and the percentages for sex and types of dementia. We calculated total scores and descriptive statistics for each health outcome at T0, T1, T2 and T3. For the POMA and CSDD, we used mean imputation for items if less than 10% was missing. The other outcomes had no missing values. Residents who participated in the POMA at T1 through T3 but not at T0 (i.e., residents with no baseline score) were excluded from the analysis. To analyze the scores of the different measures over time, we used linear mixed models with time as an independent variable and the different health outcome scores as dependent variables. The models included random intercepts for subjects to account for clustering of repeated measures within the residents. We calculated the intraclass correlation coefficient (ICC) by running intercept only models for each outcome to explore the variance explained by the grouping structure in the data. We used an AR [1] heterogeneous covariance structure for repeated measures because of the assumption that the variance was heterogeneous and that the correlations between the adjacent time points declined across measurement occasions. To interpret the estimates of fixed effects, baseline scores (T0) served as a reference. A value of *p* < 0.05 was considered statistically significant. All models included covariates to control for resident characteristics (age, sex and type of dementia).

## Results

### Participants’ characteristics

For the 55 eligible residents with dementia, legal representatives of 46 residents (84%) agreed with study participation and provided written consent. Table 1 summarizes the residents’ characteristics. At baseline, the residents’ age ranged from 66 to 99 years. Several types of dementia were identified: Alzheimer’s (33%), vascular dementia (30%), Alzheimer’s and vascular dementia combined (26%) and other types of dementia (11%), such as Parkinson’s dementia and Lewy body dementia. In total, 20 residents (43.5%) were lost to follow-up over the 9-month study period, with the most frequent reason being mortality caused by COVID-19. ICC values are all higher than 0.5, which justifies taking into account the clustering of repeated measures within subjects in the models.

**Table 1:** Characteristics of residents

Variables	Residents
Age, mean (SD)	83.2 (7.1)
Sex, female (n, %)	31 (67%)
Dementia subtype (n, %)	
Alzheimer’s	15 (33%)
Vascular dementia	14 (30%)
Alzheimer’s and vascular dementia combined	12 (26%)
Other types of dementia	5 (11%)

### Physical environment

Table 2 depicts the scores of the NHs regarding characteristics of both NHs using the OAZIS-Dementia. The NHs scored similarly on the themes of ‘privacy and autonomy’, ‘facilities’ and ‘orientation and routing’. The semi-open NH scored higher than the closed NH on the themes ‘comfort and control’ (i.e., higher light levels are used and is tailored to the type of activities taking place in the room) ‘interior’ (i.e., the building and the interior are coordinated and unified. Large spaces can be subdivided into smaller units that enhance clarity and give residents a choice) and ‘nature’ (i.e., residents can easily go outside themselves).

**Table 2:** Mean scores of the OAZIS-Dementia

OAZIS-Dementia Category	Closed NH	Semi-open NH
Privacy and autonomy	4.6	4.8
Views	3.7	5
Comfort and control	2.2	4.6
Facilities	4.8	5
Orientation and routing	3.7	3.9
Interior	2.7	4.9
Nature	3.6	5
Staff	3.7	4.7

*Note*: Mean of items on a 5-point Likert scale, on which 1 = totally disagree and 5 = totally agree. Higher scores indicate a greater likelihood of the environment having a positive effect on its residents

### Association between freedom of movement and health

**Fig. 1:**
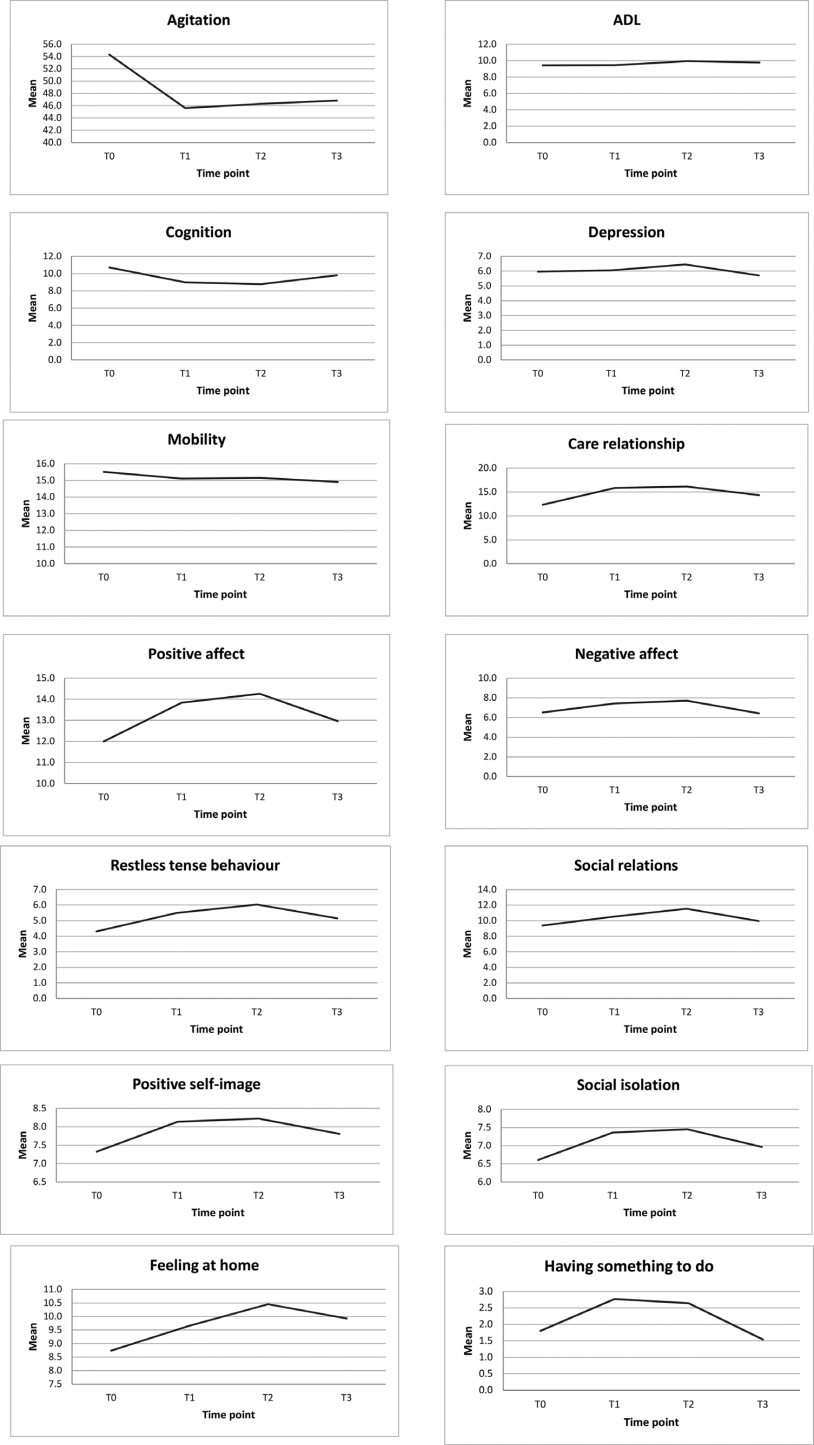
Mean scores per health outcome per time point

Mean scores for all health outcomes over time are depicted in Table 3; Fig. 1. Table 4 summarizes the results from the linear mixed models. Overall, most health outcomes improved after relocation from the closed NH to the semi-open NH. At T3, the health improvements had diminished in comparison to T2, but several scores were still elevated in comparison to T0. Scores on the quality-of-life subscales ‘care relationship’ and ‘feeling at home’ increased significantly over all time points compared to the reference time point (T0). In addition, in comparison with T0, we found a significant increase on the quality-of-life subscales ‘positive affect’, ‘negative affect’, ‘restless tense behavior’, ‘positive self-image’, ‘social isolation’ and ‘having something to do’ as well as a significant improvement in cognition at T1 and T2. For these outcomes, however, changes were no longer significant at T3. Compared to T0, the quality-of-life subscale ‘social relations’ improved significantly only at T2. We found a significant decrease in agitation levels at all time points compared to T0. POMA scores indicated a significant decrease in mobility at T1 and T2 compared to T0. Finally, no significant changes were found in ADL (Barthel) and depression (CSDD) scores.

**Table 3:** Descriptive statistics for health outcomes per time point

Questionnaires	T0				T1				T2				T3			
	N	Min–max	M	SD	N	Min–max	M	SD	N	Min–max	M	SD	N	Min–max	M	SD
Qualidem Subscales																
Care relationship	46	0–21	12.3	5.5	44	3–21	15.9	4.6	31	2–21	16.1	4.6	26	6–21	14.4	4.8
Positive affect	46	0–18	12	4.0	44	3–18	13.8	4.0	31	3–18	14,3	4.2	26	3–18	13.0	4.5
Negative affect	46	0–9	6.5	2.6	44	2–9	7.4	2.2	31	5–9	7.7	1.5	26	0–9	6.4	2.9
Restless tense behaviour	46	0–9	4.3	3.0	44	0–9	5.5	2.9	31	0–9	6.0	3.1	26	0–9	5.2	3.0
Positive self-image	46	0–9	7.3	2.5	44	2–9	8.1	2.0	31	4–9	8.2	1.4	26	1–9	7.8	2.6
Social relations	46	0–18	9.4	4.6	44	2–18	10.5	4.4	31	3–18	11.6	4.8	26	3–18	10.0	4.5
Social isolation	46	0–9	6.6	2.3	44	1–9	7.4	1.9	31	2–9	7.5	1.9	26	2–9	7.0	2.1
Feeling at home	46	0–12	8.7	3.4	44	1–12	9.7	3.0	31	3–12	10.5	2.4	26	3–12	9.9	2.8
Having something to do	46	0–6	1.8	1.8	44	0–6	2.8	2.0	31	0–6	2.7	2.2	26	0–6	1.5	1.9
CMAI	46	29–203	54.3	20.0	44	29	81	15.6	31	29–91	46.3	17.2	26	29–79	46.9	13.9
InterRai Cognition	46	0–38	10.7	3.9	44	3–15	9.0	3.1	31	4–14	8.8	2.6	26	4–16	9.8	2.8
Barthel	46	0–19	9.4	5.9	44	0–19	9.4	5.4	31	0–18	9.9	4.9	26	0–20	9.8	5.3
CSDD	43	0–20	6.0	5.1	41	0–25	6.1	5.5	30	0–18	6.5	4.6	22	1–13	5.7	3.6
POMA	24	0–28	15.5	3.6	17	9–20	15.1	3.6	14	9–19	15.1	3.1	11	9–19	14.9	3.6

N: number of participants per time point; M: mean; SD: standard deviation; Min–max represents the range of the scores per Qualidem subscales and questionnaires;

Qualidem subscales: mean of items on a 4-point Likert scale, on which 0 = never and 3 = almost every day. Negatively formulated items were recoded to 0 = every day and 3 = never. The higher the score, the higher the quality of life.

CMAI: Cohen Mansfield Agitation Inventory, mean of items on a 7-point Likert scale, on which 1 = never and 7 = several times an hour. Higher scores indicate more agitated behaviour.

InterRai Cognition: mean of items varying from 0 = independent, memory ok, absent or improved to 1 − 5 = coma, problems, present, worsened. The lower the score, the better the cognition.

Barthel: mean of items varying from 0 = dependent or incapable or incontinent to 1, 2, 3 = independent, capable or continent. 20: fully independent in basic ADL and mobility;

15–19: fairly to considerably independent; 10–14 : needs help but also does a lot by themself; 5–9 : severely in need of help; 0–4 : completely helpless

CSDD: Cornell Scale for Depression in Dementia, mean of items on a 3-point Likert scale, on which 0 = absent and 2 = severe. When an item is not assessable = 99. A value of 8 or higher indicates mild depression and of 12 or higher indicates moderate to severe depression.

POMA: Performance-Oriented Mobility Assessment, mean of items on a 3-point Likert scale varying from 0 = instable or deviation to 1, 2, 3 = stable or normal. The lower the score, the greater the mobility problem; a score lower than 19 indicates a five-fold risk of falling.

**Table 4:** Linear mixed models: analysis of health outcomes over time

	ICC^a^	Intercept			T1 (Reference is T0)			T2 (Reference is T0)			T3 (Reference is T0)		
		$\beta$	SE	95% CI	$\beta$	SE	95% CI	$\beta$	SE	95% CI	$\beta$	SE	95% CI
Qualidem subscores													
Care relationship	.55	4.3	9.5	-14.9–23.4	3.6*	.6	2.4–4.8	3.7*	.6	2.6–4.9	2.2*	.9	.3–4.0
Positive affect	.64	21.5*	8.6	4.2–39.0	1.7*	.5	.7–2.6	1.9*	.6	.7–3.1	.6	.7	− .8–2.0
Negative affect	.64	10.1*	3.9	1.9–18.3	.9*	.3	.3–1.5	1.0*	.3	.4–1.6	− .5	.5	-1.5 – .5
Restless tense behaviour	.53	.8	6.0	-11.2–12.9	1.1*	.4	.3–1.9	1.7*	.5	.6–2.8	.7	.5	− .3–1.7
Positive self-image	.74	10.5	4.2	2.0–19.1	.7*	.2	.3–1.2	.9*	.2	.4–1.3	.4	.3	− .3–1.1
Social relations	.72	18.1	9.3	− .8–37.0	1.0	.5	− .0–2.1	1.7*	.6	.4–2.9	.1	.6	-1.0–1.3
Social isolation	.68	.9	4.2	-7.7–9.4	.7*	.3	.1–1.2	.8*	.3	.2–1.3	.4	.3	− .2–1.0
Feeling at home	.50	16.1*	5.4	5.1–27.0	1.0*	.5	.1–1.9	1.8*	.5	.9–2.8	1.2*	.5	.1–2.2
Having something to do	.53	8.8*	3.5	1.8–15.8	.9*	.3	.4–1.4	.7*	.3	.1–1.3	− .5	.3	-1.1 – .2
CMAI	.69	58.3	37.0	-16.6–133.1	-8.8*	2.0	-12.8 - -4.8	-6.9*	2.1	-11.1 - -2.7	-6.5*	2.5	-11.5 - -1.4
InterRai Cognition	.60	14.2*	6.5	1.1–27.3	-1.7*	.5	-2.7 - − .7	-1.8*	.4	-2.6 - − .9	− .7	.5	-1.6 – .4
Barthel	.93	10,3	12.7	-15.5–35.9	− .2	.3	− .9 – .5	− .4	.4	-1.2 – .3	.2	.4	-1.0 – .7
CSDD	.78	4.2	11.0	-18.5–26.9	.2	.5	− .8–1.2	.7	.7	− .7–2.1	.4	.8	-1.1–2.0
POMA	.89	23.3*	9.9	2.6–44.0	− .6*	.3	-1.2 – − .0	-1.3*	.4	-2.2 - − .5	− .5	.5	-1.5 – .6

*Boldface indicates significance *P* < .05

Adjusted for age, sex and type of dementia

ICC: Intra-level Correlation Coefficient, SE: Standard Error, CI: Confidence Interval

CMAI: Cohen Mansfield Agitation Inventory, POMA: Performance-Oriented Mobility Assessment, CSDD: Cornell Scale for Depression in Dementia

## Discussion

### Main findings

This study aimed to investigate whether and to what extent increased freedom of movement is associated with the overall health of NH residents with dementia. A group of NH residents was followed over time as they moved from a closed NH to a semi-open NH, increasing their freedom of movement. The hypothesis was that more freedom of movement would relate to better health outcomes, operationalized according to the dimensions of positive health. In line with this hypothesis, we found that most dimensions of the residents’ health improved after moving from a closed NH to a semi-open NH. These health improvements did not always last until nine months after relocation. Nonetheless, none of the residents’ health scores declined over time when compared to the baseline, except for mobility scores. Moreover, a significant improvement over time lasted for agitation and the quality-of-life subscales ‘care relationship’ and ‘feeling at home’.

Although previous studies suggest that a relocation can have a negative impact on the health of NH residents with dementia [38, 39], in this study we found mostly health benefits after relocation. Several factors could mitigate the potential negative effects of a relocation, such as an improved environment in the new facility [40]. In our study, residents moved from a facility with restrictions on freedom of movement (i.e., a locked unit door) to a facility that granted them more freedom of movement, which may reflect an improved environment. This may explain the fact that as early as one month after the move, various aspects of the residents’ health had improved.

The improved health outcomes observed in this study are remarkable, as studies looking at the health trajectories of NH residents with dementia often demonstrate a decline in health outcomes [41–44]. Van der Zon et al. (2018) found a significant decrease in ‘positive affect’, ‘social relations’ and ‘having something to do’, while in our study these aspects of health improved over time. Also, the improved agitation levels until up to nine months after relocation contrasts with the course of agitation scores found in another study, where agitation scores were stable at 16 months relative to the baseline [42]. The absence of locked interior doors eliminates a potential source of agitation, which may enhance positive affect. Furthermore, in our study, depression did not significantly change over time, which is in contrast with decreased CSDD scores in other research [41, 44]. Nonetheless, in our study, the mean score on the CSDD at baseline is around 6, which indicates an overall absence of depressive symptoms. Therefore, no statement can be made about a possible association between freedom of movement and depression.

NH residents with dementia are vulnerable to mobility and functional decline due to their multiple chronic comorbidities [45], especially when they are inactive during most of the day (sleeping, watching TV or doing nothing), which could be explained by environmental aspects. When NH residents with dementia have more freedom of movement and therefore more space to move around, this may stimulate them to be more physically active [46, 47]. Despite the increased freedom of movement in this study, residents’ mobility declined over time, and their ADL remained the same. This finding is contrary to what we expected. It should be noted, however, that this study did not measure to what extent the residents used their enhanced freedom of movement in terms of physical activity. Further research is needed to explore how residents use a living environment with no boundaries and how this relates to specific aspects of health.

### Strengths and limitations

This study involved a follow-up period of nine months, which allowed us to study changes in residents’ health at multiple time points after moving to an NH with increased freedom of movement. The residents in this study were unable to self-report on their health outcomes due to their advanced stage of dementia. Nonetheless, residents’ health scores were obtained from care professionals who were closest to the residents to best reflect their actual health. Although a longer follow-up duration of one year would have allowed us to capture potential seasonal effects on health, this may have been difficult to achieve in this population. Previous research has shown that the median length of stay from NH admission to death of residents with dementia is only 5 months; around 65% percent of residents had stays of less than one year, and over 53% died within 6 months of admission [48]. Also, besides the level of freedom of movement, there are other environmental differences between the closed and semi-open NHs, which could have had an effect on the residents’ health. However, the OAZIS-dementia was designed to derive a unit-level score to describe characteristics of care environments. Larger studies in more NH units are needed to also explore how variation in unit-level factors, such as environment, impact on resident health outcomes. Additionally, due to the study design with no control group, we were unable to isolate the effect of moving to a different environment. However, as this study used the relocation of an entire nursing home unit, it was not possible to allocate residents to a control group.

Furthermore, between T1 and T2 all residents with dementia in our study were infected by COVID-19 and a relatively large proportion of residents died. The disease, next to the measures to prevent or contain COVID-19 outbreaks, may have negatively impacted the residents’ health outcomes. Thus, it is possible that the findings would have been more positive in the absence of COVID-19.

In this study, the scores on the CSDD and POMA were registered in the care record of residents. It is unknown whether this information was used in the residents’ treatment or care, for example, by adjusting the daily activities or providing physical training. If this was the case, it may have affected scores in follow-up measurements. Also, because there is a lack of reported psychometric information on the POMA, further psychometric testing of this instrument is needed.

Finally, there is no validated questionnaire that measures all six dimensions of positive health. Therefore, we self-selected separate questionnaires for each dimension, which were developed and validated specifically for NH residents with dementia. However, these separate questionnaires may not have measured the six dimensions of positive health in a decisive way. In addition, because a questionnaire regarding existential health was lacking, we were not able to measure the impact of freedom of movement on this dimension of positive health. Recently, a new measurement tool for positive health among the general population has been developed and validated in the Netherlands [49–51]. We recommend further developing this tool and validating it among (proxies of) NH residents with dementia.

### Implications for future research and practice

The results of this study support the hypothesis that increasing NH residents’ freedom of movement is associated with improved health outcomes. Despite these health benefits, in practice the dilemma of weighing safety and freedom often still leads to restricted freedom of movement for NH residents with dementia [10, 17]. Dutch legislation states that freedom of movement must be granted unless there are strong arguments to limit a person’s freedom [52]. In many NHs, this requires a culture change towards providing a living environment with freedom of movement as a starting point. To support NHs in achieving this, we need a better understanding of physical and social environmental factors that may hinder or facilitate the implementation of freedom of movement for residents with dementia. For example, a study among formal care professionals in nursing homes showed that surveillance technology was regarded as a way to provide more freedom to residents [53], yet care professionals have also expressed concerns such as the violation of privacy of residents and failing technology. More education for professionals and improved technology might increase the use of surveillance technology and, as such, facilitate the provision of freedom of movement to NH residents. Such insights could be used to optimize the implementation of freedom of movement and ultimately improve the health of NH residents.

## Conclusion

This study has shown that increasing the freedom of movement of NH residents with dementia is associated with an improvement in different dimensions of health over time. These findings add to the growing evidence supporting the benefits of more freedom of movement on the overall health of NH residents with dementia.

## Data Availability

The dataset generated and/or analyzed during the current study is not publicly available due to the sensitive nature of the data but is available from the corresponding author upon reasonable request and after approval by the Ethics Review Board of Tilburg University.

## Abbreviations

NH: Nursing home
ADL: Activities of Daily Living
CMAI: Cohen-Mansfield Agitation Inventory
CSDD: the Cornell Scale for Depression in Dementia
InterRAI LFTC: InterRAI Long-Term Care Facilities System
POMA: Performance-Oriented Mobility Assessment according to Tinetti
ICC: intraclass correlation coefficient
N: number of participants per time point
M: mean
SD: standard deviation
SE: standard error
CI: confidence Interval
